# Safety of concomitant tranylcypromine treatment during electroconvulsive therapy (ECT) series

**DOI:** 10.1192/j.eurpsy.2022.671

**Published:** 2022-09-01

**Authors:** E. Kavakbasi, G.M. Ciftci, M. Tonkul, B. Baune

**Affiliations:** University Hospital Muenster, Department Of Psychiatry, Münster, Germany

**Keywords:** Electroconvulsive therapy, ECT, Tranylcypromine, MAOI

## Abstract

**Introduction:**

Tranylcypromine (TCP), an irreversible monoamine oxidase inhibitor (MAOI), is recommended for difficult-to-treat depression. Besides the requirement of a low-tyramine diet, there are concerns about the safety of TCP treatment during anaesthesia and electroconvulsive therapy (ECT). For safety reasons, many psychiatrists prefer to terminate TCP before ECT.

**Objectives:**

To assess the safety of tranylcypromine treatment during ECT series in patients with difficult-to-treat depression (DTD).

**Methods:**

In this retrospective study we report on n=19 patients, who were treated with tranylcypromine during the ECT series. ECT parameters, clinical and safety data were obtained from our clinical database.

**Results:**

Mean age of patients was 51 years (range 29-77) at time of the first ECT sessions. 58 % (n=11) of patients were female. In total, 198 ECT sessions were analysed (mean 11, median 9,5 per patient). Mean TCP dose was 44 mg at time of first ECT (median 43). Concomitant TCP and ECT treatments were well tolerated during the entire ECT series. In one case TCP treatment was discontinued due to self-limiting bigeminus during the ECT session. In another case TCP and other drugs as well as the ECT series were stopped after the patient developed delirium. At the end of ECT series the mean TCP dosage was 37 mg.

**Conclusions:**

Tranylcypromine appears to be safe during ECT series and does not necessarily have to be terminated prior to electroconvulsive therapy.

**Disclosure:**

No significant relationships.

